# Preparation of Terbinafin-Encapsulated Solid Lipid Nanoparticles Containing Antifungal Carbopol^®^ Hydrogel with Improved Efficacy: In Vitro, Ex Vivo and In Vivo Study

**DOI:** 10.3390/pharmaceutics14071393

**Published:** 2022-06-30

**Authors:** Nilesh R. Rarokar, Sunil S. Menghani, Deweshri R. Kerzare, Pramod B. Khedekar, Ashish P. Bharne, Abdulhakeem S. Alamri, Walaa F. Alsanie, Majid Alhomrani, Nagaraja Sreeharsha, Syed Mohammed Basheeruddin Asdaq

**Affiliations:** 1 Computer Aided Drug Design Laboratory, Department of Pharmaceutical Sciences, Mahatma Jyotiba Fuley Shaikshanik Parisar, Rashtrasant Tukadoji Maharaj Nagpur University, Amravati Road, Nagpur 440033, India; nileshrarokar@outlook.com (N.R.R.); pbkhedekarudps@gmail.com (P.B.K.); ashishbharne2@gmail.com (A.P.B.); 2 Department of Pharmaceutical Chemistry, Krupanidhi College of Pharmacy, Bangalore 560035, India; 3 Department of Pharmaceutical Chemistry, Dadasaheb Balpande College of Pharmacy, Nagpur 440037, India; kerzarepritee@gmail.com; 4 Department of Clinical Laboratory Sciences, The Faculty of Applied Medical Sciences, Taif University, Taif 21944, Saudi Arabia; a.alamri@tu.edu.sa (A.S.A.); w.alsanie@tu.edu.sa (W.F.A.); m.alhomrani@tu.edu.sa (M.A.); 5 Centre of Biomedical Sciences Research (CBSR), Deanship of Scientific Research, Taif University, Taif 21944, Saudi Arabia; 6 Department of Pharmaceutical Sciences, College of Clinical Pharmacy, King Faisal University, Al-Ahsa 31982, Saudi Arabia; sharsha@kfu.edu.sa; 7 Department of Pharmaceutics, Vidya Siri College of Pharmacy, Off Sarjapura Road, Bangalore 560035, India; 8 Department of Pharmacy Practice, College of Pharmacy, AlMaarefa University, Dariyah, Riyadh 13713, Saudi Arabia

**Keywords:** solid lipid nanoparticles, hydrogel, antifungal, terbinafin, *Candida albicans*

## Abstract

The present research was aimed to develop a terbinafin hydrochloride (TH)-encapsulated solid lipid nanoparticles (SLNs) hydrogel for improved antifungal efficacy. TH-loaded SLNs were obtained from glyceryl monostearate (lipid) and Pluronic^®^ F68 (surfactant) employing high-pressure homogenization. The ratio of drug with respect to lipid was optimized, considering factors such as desired particle size and highest percent encapsulation efficiency. Lyophilized SLNs were then incorporated in the hydrogel prepared from 0.2–1.0% *w/v* carbopol 934P and further evaluated for rheological parameters. The z-average, zeta potential and polydispersity index were found to be 241.3 nm, −15.2 mV and 0.415, respectively. The SLNs show a higher entrapment efficiency of about 98.36%, with 2.12 to 6.3602% drug loading. SEM images, XRD and the results of the DSC, FTIR show successful preparation of SLNs after freeze drying. The TH-loaded SLNs hydrogel showed sustained drug release (95.47 ± 1.45%) over a period of 24 h. The results reported in this study show a significant effect on the zone of inhibition than the marketed formulation and pure drug in *Candida albicans* cultures, with better physical stability at cooler temperatures. It helped to enhance skin deposition inthe ex vivostudy and improved, in vitro and in vivo, the antifungal activity.

## 1. Introduction

Fungal infections of the skin, scalp, or nails are treated by oral and topical antifungal agents. Terbinafin hydrochloride is a widely used drug for the treatment of fungal infections of the skin, and it has been well tolerated by oral or topical administration [1]. However, insufficient bioavailability and hepatic first pass metabolism (about 40%) made the topical route more suitable for drug administration [2]. It offers and enables a reduction in the systemic load as well as systemic side effects of the active pharmaceutical ingredients (API) [3]. Controlled drug delivery systems are one of the best approaches that have the potential to cure disease conditions by targeting specific cells in the body [4]. Thus, site specificity and time specificity are the advantages of nanotechnology, a novel drug delivery system [5]. Nanoparticles have proven their potential to target the cells or receptors at the site of action. Hence, this can be helpful to overcome the pharmacokinetic drawbacks of drugs [2].

Solid lipid nanoparticles act as a carrier for the delivery of therapeutic agents. SLNs are generally fabricated by the incorporation of a solid form of lipid into the o/w type of emulsion with the help of a stabilizer, which results in the easier entrapment of API. SLNs are composed of physiological supermolecules, and hence the pathways for lipid transportation and metabolism are already gifted within the body to confirm thein vivofate of the carrier. These are stable for an extended period and straight forward to rescale compared to different mixture systems, so they are also vital for several modes of targeting. SLNs contain a variety of drugs from various pharmacological categories, such as steroids, vitamins, cancer-fighting agents, and antifungals [6,7,8,9,10,11,12]. Since SLNs are speculated to deliver drugs to the site of action [13], they are also widely used for topical applications. These SLN formulations additionally supply higher localization, occlusiveness, controlled unleash, and skin association for higher effectivity [14].

Previously, Vaghasiyaet al.prepared TH-loaded SLNs by the solvent-injection method, but the SLNs with the desired entrapment efficiency and lower particle size were not obtained [15]. Then the SLNs were supplied in carbopol gel, which shows improved skin retention ability upon topical application to the abdominal skin. In another study, Chenet al.manufactured SLNs by the microemulsion technique using glyceryle monostearate (GMS) and glyceryle behenate. The combination of both these lipids enhanced the skin penetration of TH across the dermal layers and resolved the practical problem of a longer administration period [16]. All this literature revealed that incorporation of GMS as a lipid for SLNs preparation helps to improve the skin penetration, whereas SLNs incorporation in carbopol gel enhances the skin retention on the skin. Hence, the present study was designed to prepare a TH-loaded SLNs carbopol gel to enhance the skin retention with improved skin penetration upon topical application for the treatment of fungal infection. Terbinafine is a standard agent that structurally belongs to the allylamine category and pharmacologically acts by selective inhibition of plant life squalene epoxidase. It is a broad-spectrum agent having associated activity against yeast, fungi, molds, and dermatophytes [17]. The formulation of TH is difficult on the bench, since it is very lipophilic (log *p* value of 3.3) and nearly insoluble in water. Topical application of TH has the advantages of direct distribution and targeting ability to the affected area of the skin, as well as low dose requirements and minimal toxicities [18].

## 2. Material and Methods

### 2.1. Drug and Chemicals

Terbinafine hydrochloride was obtained from FDC LIMITED, (Waluj, Aurangabad India) as a gift sample. Colorcon Asia Pvt. Ltd., (Verna, Goa, India) provided glyceryl monostearate (GMS), Compritol 888 ATO, and Precirol ATO5. The Hi-Media Lab. Pvt. Ltd., Mumbai, (India) provided sabouraud dextrose agar (SDA), chitosan, and pluronic F68. Using a Milli-Q system, pure water was obtained (Millipore, Billerica, MA, USA). All of the other compounds were of the analytical variety.

### 2.2. Drug and Excipient Compatibility Studies

#### 2.2.1. Thermal Analysis

Differential scanning calorimetry (DSC) was used to examine the drug, polymers, and their physical mixture with TH. DSC measurements were carried out in open pans using a DSC Q20 (Mettler-Toledo, Zurich, Switzerland) with a sample size of approximately 5 mg, weighed in each aluminum pan. From 0 to 400 °C, samples were gradually heated at a rate of 10 °C per minute. Nitrogen was supplied at a flow rate of 40 mL/min as a purging gas. Universal Analysis software version 4.5 A, build 4.5.0.5, was used to examine the data and dates acquired (TA Instruments, Inc., New Castle, DE, USA). The procedure was repeated for the freeze-dried SLNs.

#### 2.2.2. Fourier Transform Infrared (FTIR) Spectroscopy

The possible interaction of a physical mixture of drug and polymer was investigated using the FTIR spectroscopy technique. The samples were dried for 2 h in a hot air oven at 50 °C. The samples were crushed and completely mixed with potassium bromide at a ratio of 1:100 (Sample:KBr), and circular KBr discs were formed at a pressure of 10 t/nm^2^ by compression. The IR spectra obtained were compared and studied to detect, the physical interactions between the drug and the polymers, if any.

### 2.3. Preparation of Solid Lipid Nanoparticles (SLNs)

Solid lipid nanoparticles (SLNs) containing TH were prepared by the hot high-pressure homogenization method reported previously [19]. In brief, glyceryl monostearate (GMS) used as a lipid phase was melted by heating in a water bath at 80 °C. About 1.0–3.0% *w/w* TH was added to the molten lipid phase simultaneously. The aqueous solution of pluronic F68 was prepared separately and was dispersed in the previously prepared molten lipid phase at 80 °C with continuous stirring on a magnetic stirrer (Remi Instruments Ltd., Mumbai, India) at 400 RPM for 30 min to get a pre-emulsion. The prepared pre-emulsion was subjected to homogenization using a high-pressure homogenizer (PANDA 2K, Niro Soavi, Parma, Italy) at 500 bar pressure for up to 5 cycles to form the SLN dispersion. The prepared SLN dispersion was allowed to cool at normal conditions and was subject to characterization.

### 2.4. Characterization of SLNs

#### 2.4.1. Particle Size, Polydispersity Index and Zeta Potential Measurement

Photon correlation spectroscopy (PCS) was used to determine the particle size of the blank and TH-loaded SLNs using dynamic light scattering on a Zetasizer^®^ nano (Model: Zen 3600, Malvern Instruments, Malvern, UK) aided by a 5-mW helium neon laser (wavelength output of 633 nm). The experiment was carried out at a temperature of 25 °C and a 173° angle [20]. On the instrument, a run time of at least 40–80 s was set. As a dispersion medium, water was used. The polydispersity index was also used to investigate the nanoparticle dispersion. Smoluchowski’s equation was used to calculate the zeta potential from the electrophoretic mobility using a previously described approach [21]. All results were obtained in triplicate.

#### 2.4.2. Percent Drug Loading

About 1 mL of the SLN dispersion was taken into pre-weighed vials, allowed to freeze dry, and then dissolved in methanol [22]. The concentration of TH present in the solution was estimated by spectrophotometry at 283 nm. The below formulae were applied to calculate the drug-loading capacity (DL):

$$\%\;Drug\;Loading=\frac{Mass\;of\;drug\;in\;SLNs}{Mass\;of\;SLNs\;containing\;drug\;}\times 100$$

#### 2.4.3. Entrapment Efficiency

The entrapment efficiency (EE), or the amount of TH contained in SLNs, was assessed using a variety of methods previously described [23]. One milliliter of SLNs containing TH was put into a Centricon^®^ reservoir (Model: YM-100, Amicon, Millipore, Bedford, MA, USA). The dispersion of SLNs was centrifuged at 15,000× *g* rpm for 40 min. Filtration was used to eliminate the free TH component. Dilution with wood spirit (methanol) was carried out for the dispersed filtrate, followed by TH content determination using HPLC to compute the total concentration of TH (C_t_) and the concentration of TH retained in the filtrate post centrifugation (C_f_). The entrapment efficiency was determined using the following equation:

$$\%\;EE=\frac{Amount\;of\;drug\;\left(C_{t}\right)-Amont\;of\;drug\;in\;supernatant\;\left(C_{f}\right)}{Amount\;of\;drug\;added}\times 100$$

### 2.5. Preparation of Lyophilized SLNs

The SLN dispersions were rapidly frozen at −75 °C along with the mannitol as a cryoprotectant in varying concentrations, such as 2, 4, 6, 8 and 10% *w/v* in a deep-freezer for 1 h. The mannitol-containing SLN dispersions were then freeze dried (Freeze Drier, VirTis Benchtop SP Industries, Warminster, PA, USA) for 72 h by applying a vacuum at 100 mTorr. Freeze-dried SLN powder was then collected and used for further analysis.

### 2.6. Characterization of Lyophilized SLNs

#### 2.6.1. Scanning Electron Microscopy (SEM)

SEM (JSM-6390LV, JEOL, Tokyo, Japan) was used to examine the morphological structures of the TH and optimized freeze-dried SLNs formulation [24]. A freeze-dried SLNs formulation that had been optimized was put on double-sided carbon tape and placed on a brass stub. Using the auto fine coater, a thin layer of metal palladium was applied to the surface powder (Model: JFC1600, Jeol Ltd., Tokyo, Japan). A scanning electron microscope (Model: JSM-6390LV, Jeol Ltd., Tokyo, Japan) attenuated with a digital camera was used to examine the metal-coated samples at a 10 kilovolt accelerating voltage.

#### 2.6.2. X-ray Diffraction Analysis (XRD)

The changes in polymorphic form and crystalline nature of the drugs after the formation of SLNs were studied by an XRD. The XRD patterns of the samples (API, physical mixture of API and lipid, freeze-dried SLNs formulation) were estimated by an X-ray diffractometer (BruckerAxs, D8 Advance, Karlsruhe, Germany) provided with a source of radiation, a Cu-Kα line, operated at a 40 kV voltage and a current of 30 mA. Each sample was analyzed in the 2θ angle range between 100 and 600. The estimation was carried out at a scanning rate of 30/min and a step size of 0.020 was maintained.

### 2.7. Formulation of the TH-Loaded SLNs Hydrogel

The hydrogel of TH-loaded SLNs was prepared by using different gelling agents, such ascarbopol 934P, chitosan, and pluronic F127 [25]. The result of a preliminary study shows that carbopol 934P (0.2–1.0% *w*/*v*) and chitosan (0.5–2.0% *w*/*v*) were found to be compatible with TH-loaded lyophilized SLNs. Carbopol 934P showed greater ease of spreadability than chitosan. Hence, carbopol 934P was used for the preparation of the gel and chitosan was used as gelling agent to improve the gel strength. Different concentrations of carbopol 934P were dispersed using a mechanical stirrer at a speed of 800 rpm for a 3 h duration in order to improve the stiffness of the gel with optimum viscosity. Triethanolamine (0.05% *w*/*w*) was added with the aid of a magnetic stirrer to neutralize the gelling system. Phenyl mercuric nitrate (0.02% *v*/*v*) was added as a preservative to prevent it from microbial contamination.

### 2.8. Characterization of the TH-Loaded SLN-Based Hydrogel

#### 2.8.1. Measurement of Viscosity and pH

The apparent viscosity and rheological behavior of the TH-loaded SLN-based gel was measured using a Brookfield Viscometer provided with spindle no. 5 at 10 to100 rpm [26]. A pH meter (Elico Pvt. Ltd., Hydrabad, India) was used to measure the pH of the gel formulations by dissolving one gram of the TH-loaded SLN-based gel in 100 mL of distilled water. The pH meter was calibrated with buffered solutions of pH 4.0 and 7.0 before measuring the pH of the samples.

#### 2.8.2. Extrudability and Spreadability

The formulations were crammed in the collapsible tubes. The extrudability of the formulation was estimated, which is explained in terms of weights in grams required to extrude a 0.5 cm ribbon of gel in 10 s.

The spreadability represents the extent of area to which a gel readily spreads, once applied on the affected part. The spreadability confines the therapeutic efficacy of a formulation. The Wooden block and glass slide apparatus was employed to check the spreadability of the TH-loaded SLN-based gel [27]. This is expressed in terms of the time (seconds) taken by the two slides to slip off from the gel and be placed in between the slides under the direction of a certain load. Separation of the two slides shall be in the minimum time and as low as possible, which is better for a good spreadability of the gel. About 95 g of gel was placed to the pan and the time required to separate the upper slide (movable) completely from the fixed slides was noted. It was calculated by using the formula S = M × L/T, where M is the weight tied to upper slide, L is the length of glass slides, and T is the time taken to separate the slides.

#### 2.8.3. Determination of Gel Strength

The gel strength test was performed using the gel strength apparatuses, using a modified method previously reported by Yong et al. [28]. About 50 g of TH-loaded SLN-based gel was transferred into a 100 mL measuring cylinder and a piston (weighing about 35 g) was then placed onto the surface of the gel. The gel strength was measured as the time (seconds) required for moving the piston by 5 cm through the gel. If necessary, various weights were placed when more than 5 min were taken to drop the apparatus into the gel.

### 2.9. Determination of Drug Content

A 100 mL volumetric flask [29] was used to properly weigh 200 mg of TH-loaded SLN-based hydrogel before dilution with methanol and 45 min of sonication. Following sonication, 5 mL was pipetted out and diluted with methanol once more, this time up to 50 mL. Finally, the average values of three separate measurements of absorbance at 283 nm were calculated.

### 2.10. In Vitro Drug Release

An in vitro drug release study of the TH-loaded SLN-based hydrogel was performed using Franz diffusion [30]. The dialysis membrane (Himedia^®^ Pvt. Ltd., Mumbai, India) has a cut-off molecular weight of 12,000–16,000 Dalton and a pore size of 2.4 nm and is placed between the donor and receptor compartments. This membrane allows passage of drugs to the receptor compartment from the donor compartment. Phosphate-buffered saline (PBS), pH 7.4, was filled in the receptor compartment. The whole assembly was shaken on a magnetic stirrer at a speed of 100 rpm and a temperature of 37 ± 0.5 °C. Samples were periodically withdrawn from the receptor compartment and absorbance was measured at 283 nm. The volume of sample withdrawn was replenished each time with the same volume of fresh PBS. The results were taken in triplicates and the cumulative percent drug release was calculated and plotted against the time.

### 2.11. ExVivo Skin Permeation Study

The exvivo permeation study of the TH-loaded SLN-based gel was carried out on abdominal skin of Sprague-Dawley rats using the procedure reported previously [31]. The hair and subcutaneous tissues were removed using a shaving razor. The dermis side was wiped with isopropyl alcohol to get rid of the residual fat material, washed with distilled water, and stored under pH 7.4 PBS in a freezer till further use. The Franz diffusion cell with an efficient and effective diffusion area of 3.14 cm^2^ was used for this study. The skin was placed over the diffusion cell along with the dermal side to bear with the receptor phase. Diffusion medium of 40 mL (pH 7.4 PBS) was filled in the receptor compartment and was then subjected to pausing to be stirred less, at 100 rpm, using a magnetic stirrer. The equilibrium of the system was allowed to sustain at 37 ± 0.5 °C using a water bath. About 100 mg of gel was applied and spread over the diffusion area on the skin. The sample solution, for estimation, was taken out at predetermined time intervals (0.5 to 14 and 24 h) from the receptor compartment and replenished with the fresh buffer medium. Every study was continued for a day (24 h) and the percent drug released across the skin was calculated.

### 2.12. Evaluation of Antifungal Efficacy of Formulation

#### 2.12.1. In Vitro Antifungal Activity

In vitro antifungal activity was measured by the “cup plate method” by using Sabouraud dextrose agar (SDA), a medium for the cultivation of *Candida albicans,* and the previously sterilized antifungal assay agar medium. In brief, a quantity of fungal culture equal to 1 mL was inoculated and thoroughly mixed with the antifungal assay agar media. The agar medium was left to solidify in the Petridish and 4 holes were made by a cork borer having a diameter of 1 cm. A quantity of gel formulation equal to 30 mg was poured into 2 holes, and the remaining 2 holes were sampled with a marketed gel formulation. It was subjected to diffuse at 25–27 degrees Celsius for an hour. The plates were placed in an incubator at 25 °C. The diameters of the growth zones of inhibition were measured every 24 h for 3 days.

#### 2.12.2. In Vivo Antifungal Activity

The in vivo antifungal activity of the produced mixture was determined using a *C. albicans*-induced mycosis model in Wistar albino rats (100–150 g) [32]. The entire protocol was followed with the agreement of the Institutional Animal Ethics Committee. To begin, the rat’s hair was removed using a hair removal treatment (depilation). The skin was marked on a 2- to 3-cm^2^ region, and then the skin was scraped out somewhat the next day using sandpaper. A glass rod was used to apply a previously prepared *C. albicans* inoculums. Three groups of Wistar albino rats were formed, each with five animals. The first group was designated as a control group that received no therapy. A commercially available terbinafine gel was provided to the second group. The third set of animals received the test formulation. Except for the first group, animals were treated for 6 days after infection. Animals were observed for any obvious morphological alterations. After six days, the animal skin was cleaned with a cotton swab. Skin was removed from the treatment area and homogenized in a tissue homogenizer with 5 mL saline. After streaking on a solid yeast extract–peptone–dextrose medium, the homogenate was incubated at 25 °C for 4 days. The number of colony-forming units (CFUs) on the agar plate was counted, and the logarithm of the number of CFUs per infected site was determined. A positive animal was defined as one that had more than one fungal colony.

### 2.13. Stability Study

The lyophilized SLNs and TH-loaded SLN-based gel were stored in airtight containers and further subjected to evaluation of physical stability during storage conditions according to International Conference on Harmonization (ICH) Q1A (R2) guidelines (FDA, 2003). The particle size as well as percentage of drug content were also determined periodically to check the physical stability of the formulations.

An accelerated stability study for the TH-loaded SLN-based gel was also carried out for 3 months. In the first test, the TH-loaded SLN-based gel was packed in a collapsible aluminum tube and kept at 25 °C and a humidity of 60%. Secondly, another sample of the formulation was kept at a freezing temperature (Remi Instruments Ltd., Mumbai, India). After a predetermined time interval, the TH-loaded SLN-based gel was evaluated by measuring the pH and determining the drug content.

### 2.14. Statistical Analysis

The mean and standard deviation were used to express all of the data (SD). GraphPad^®^ Prism^®^ software version 5.03, Dr. Harvey Motulsky, San Diego, USA, was used to conduct the statistical analysis, which included a two-way analysis of variance (ANOVA) and a Bonferroni post-test (San Diego, CA, USA). If the *p* value was less than 0.05, the differences between the means were judged to be significant.

## 3. Results and Discussion

### 3.1. Drug and Excipient Compatibility Study

#### 3.1.1. Thermal Analysis

**Figure 1:**
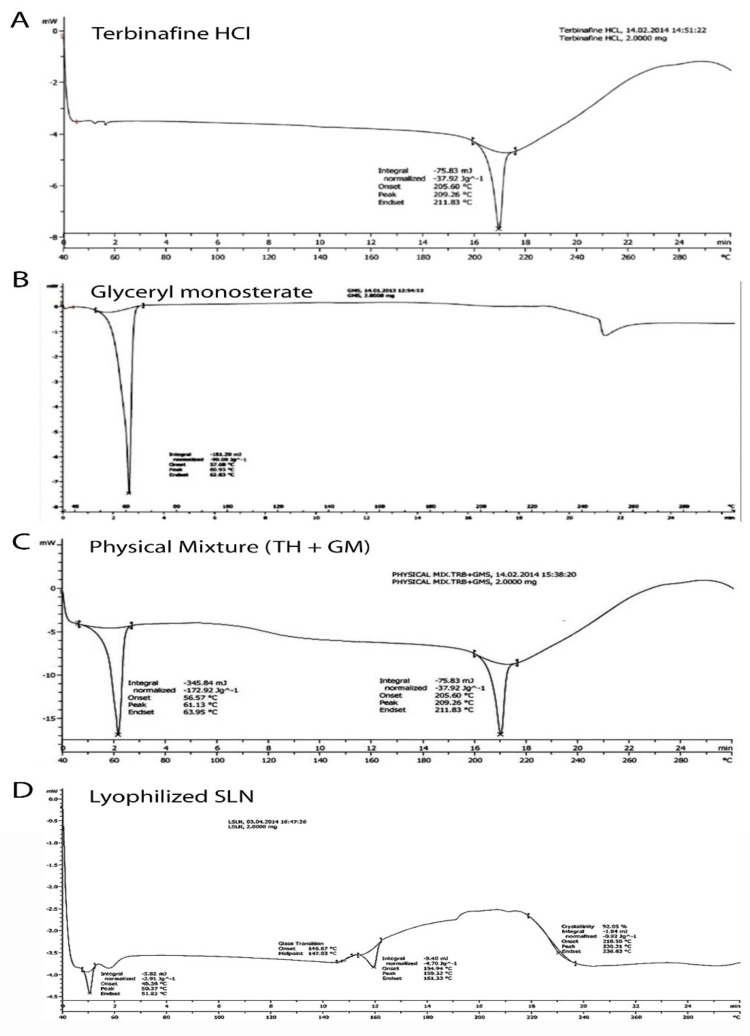
Differentialscanning calorimetric thermograms showing the endothermic peak of terbinafin hydrochloride (**A**); glyceryle monostearate (**B**); physical mixture of terbinafin hydrochloride (TH) and glyceryle monostearate (GM) (**C**); and lyophilized solid lipid nanoparticles (**D**).

The DSC thermograms of pure TH (A), glyceryl monostearate (B), physical mixture of TH, as well as GMS (C) and lyophilized SLNs (D) are represented in Figure 1. TH showed a characteristic crystalline form melting peak at about 209.26 °C and the DSC curve of glyceryl monostearate reflected one endothermic peak at 60.93 °C. The SLNs dispersion showed a small endothermic peak around 230.31 °C. The DSC curve of TH showed a characteristic crystalline form melting peak at about 209.26 °C and the DSC curve of glyceryl monostearate showed one endothermic peak at 60.93 °C. These results revealed the absence of an interaction between the drug and lipid excipient. Previous research [33] interpreted similar types of findings [33]. The SLNs dispersion showed a small endothermic peak around 230.31 °C. This change in melting point is indicative of the transformation of the crystalline form to the amorphous form of a drug.

#### 3.1.2. Fourier Transform Infrared (FTIR) Spectroscopy

**Figure 2:**
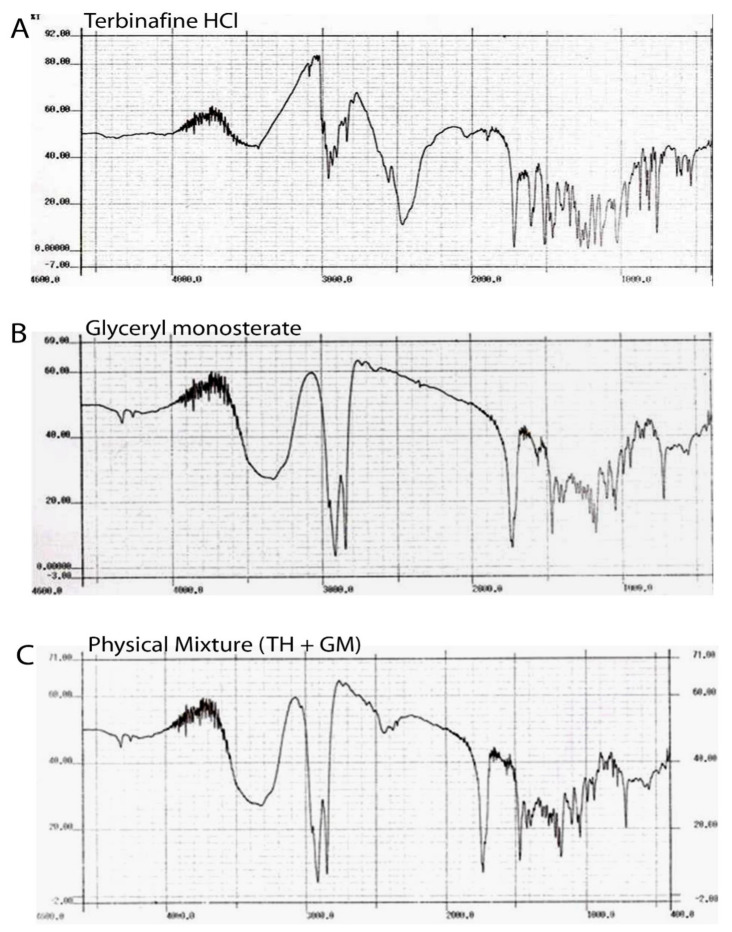
FTIR spectrum of pure TH (**A**), glyceryl monostearate (**B**), physical mixture of TH and GMS (**C**).

Figure 2 illustrates the FTIR spectrum of pure TH (A), glyceryl monostearate (B), physical mixture of TH, GMS (C), and lyophilized SLNs (D). All the characteristic peaks obtained at wave numbers 2400, 1700, 1400, and 850 cm^−1^ were fromthe drugs and are present in the spectrum of a formulation similar to that of TH. The peaks found in the physical mixture of TH and GMS were present at the same wave number as those of the individual peaks of TH and GMS. Thus, no interaction of the drug with a lipid can be concluded.

### 3.2. Characterization of SLNs

#### 3.2.1. Particle Size, Polydispersity Index, and Zeta Potential

The data are presented in Table 1. The particle size of the SLNs was found to be in the range of 241.3 to 321.8 nm. Particle size analysis revealed that the increase in the size of the particles is directly proportional to the increase in the amount of lipid [34].

The polydispersity index was found in the range of 0.415 to 0.577. The lower values of the polydispersity index correspond to the wide distribution of particles.

The zeta potential values of all batches were found within the range of −15.2 mV to −24.8 mV. The value of the zeta potential from −15.0 mV to −30.0 mV indicates that the dispersions remain deflocculated owing to electrostatic repulsion between the particles and are physically stable over time [35,36]. The zeta potential was influenced by the anionic property of the lipid matrix and thus reflects the physical stability of the SLNs [37].

#### 3.2.2. Percent Drug Loading and Encapsulation Efficiency

The data are presented in Table 1. The drug-loading percentage was found in the range of 2.12 to 6.3602. The TH-loaded lyophilized SLNs prepared using the high-pressure homogenization technique showed a higher entrapment efficiency, which is about 98.36%. A similar type of result was reported previously [38].

### 3.3. Characterization of Lyophilized SLNs

#### 3.3.1. Scanning Electron Microscopy

**Figure 3:**
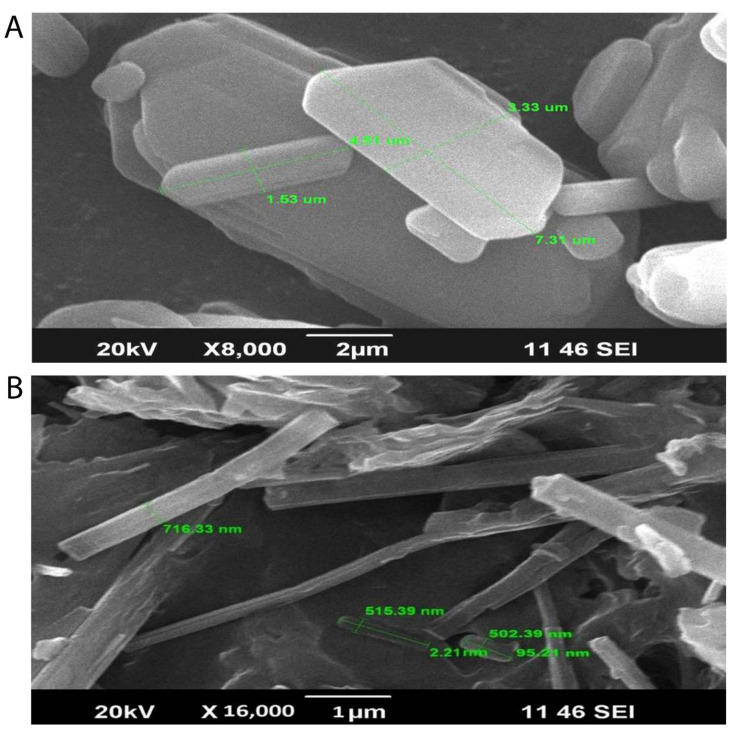
Scanning electron microscopy images showing the hexagonal crystalline form of TH (**A**) with varying sizes of particles and longitudinal amorphous structures, which confirm the formation of SLNs (**B**).

Scanning electron microscopy photographs of TH and the optimized batch of SLNs (PF1) are presented in Figure 3. The hexagonal crystalline form of TH (Figure 3A) was observed on the scanning electron microscope images, with varying size of particles. Figure 3B illustrates the longitudinal amorphous structures, which confirm the formation of SLNs.

The varying particle size was obtained during the preparation of the SLNs, as the previous literature reported the increased in particle size while usingcompritol and pluronic f68 as a surfactant, and there were almost a 4-fold increase in particle size observed. This increase in particle size leads to the increased viscosity of the lipid matrix. On the other hand, the incorporation of a higher amount of drugs could be the reason for the significant increase in viscosity, which directly relates to the increase in particle size of the SLNs [39,40,41]. However, the size shown in the figure is the dimension of the particles and it is not considered as an average mean diameter of the particles. The mean diameter of the SLNs was found in the range of 241.3 to 321.8 nm.

#### 3.3.2. X-ray Diffraction Studies

**Figure 4:**
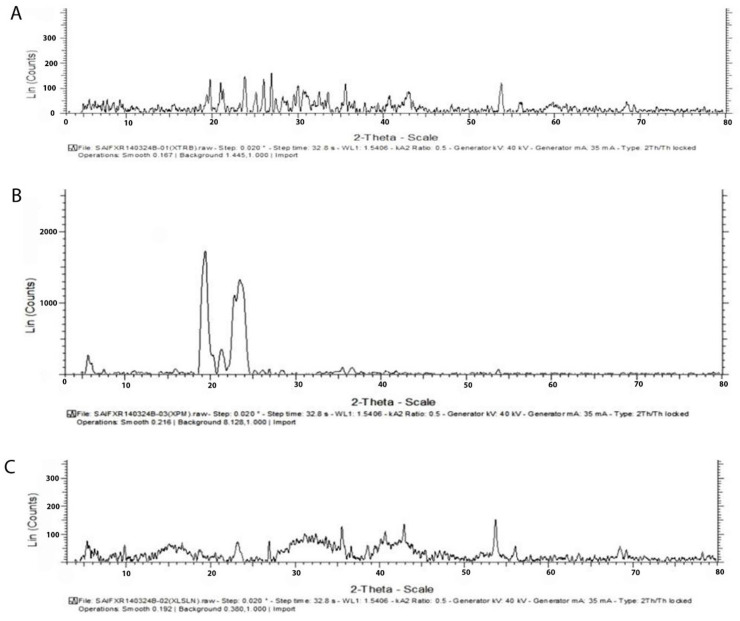
X-ray diffractogram of TH shows sharp peaks at diffraction angles (2θ) with the typical crystalline pattern (**A**), csrystalline pattern of physical Mixture of glyceryl monosterate (**B**) and Freezed Dried SLNs preparation (**C**).

In Figure 4, X-ray diffraction patterns of TH, physical mixture bearing the TH along with the glyceryl monostearate, and the freeze-dried SLNs preparation are presented. The X-ray diffractogram of TH shows sharp peaks at diffraction angles (2θ) of 19.656°, 20.914°, 23.750°, 25.032°, 29.948°, 30.688°, 35.508°, 37.780°, 42.921°, 53.747°, and 56.013°, which confirms the typical crystalline pattern. However, all the major characteristic crystalline peaks become visible in the diffractogram of the nanoparticulate system but with low intensity. Thus, it confirms that some amount of the drug is converted into its amorphous form.

The results of the X-ray diffractometry analysis were supported by FTIR and DSC studies.

These results collectively indicated that the crystallinity of the drug decreased when formulated as the SLNs using lipids.

Further, they suggested the conversion of TH from crystalline to molecular form.

### 3.4. Characterization of the TH-Loaded SLN-Based Hydrogel

#### 3.4.1. Rheological Behavior

**Figure 5:**
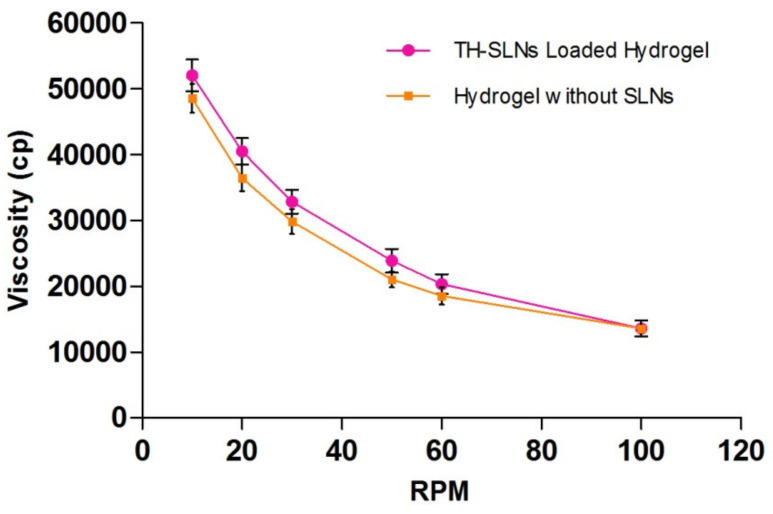
Formulations showed pseudo-plastic flow behavior. Rheological behaviors of the TH-loaded SLNs containing the carbopol hydrogel, showing no coincidence of the up curve with the down curve, indicating non-Newtonian thixotropic behavior.

The formulation showed pseudo-plastic flow behavior as results revealed in Figure 5. The SLN-loaded hydrogel revealed a distinct up and down curve; that is, the samples were non-Newtonian in nature, with a thixotropic behavior. The formulated hydrogel takes time to come back to its original viscosity state after application of shear stress but during this process it maintained its structural properties associated with the gel network [42]. Rheological and texture analyses showed that the TH-loaded SLN-based gel had satisfied the ideal rheological and texture properties in order to assist its topical application.

#### 3.4.2. Determination of Viscosity, pH, Gel Strength, Spreadability, Extrudability and Drug Content

These gel formulation characteristics reflect improved patient compliance for topical application. The formulation was subjected to the determination of these values (*n* = 6), and the mean ± SEM for the viscosity of the TH-loaded SLN hydrogel is presented in Table 2.

It was free from any particles, easily spreadable, extrudable, and followed a pseudoplastic flow. The optimized SLNs were then incorporated into the Carbopol 934P gel base with tri-ethanolamine as a neutralizer and phenylmercuric nitrate as a preservative. The formulated gel appeared white in color and was acceptable. The pH of the formulated gel was found within the normal pH range of the skin (pH~5.5 to 6.5), which is under the range (pH 3–9) essentially required for treatment of skin infections [43].

Gel strength is an important parameter for topical gels. It indicates suitability for easy application and skin retention without leakage after application. Our formulation showed better gel strength, and that was achieved with 0.6% Carbopol 934P. Spreadability dictates the ease of application of a gel onto the skin’s surface [44]. The formulated gel showed spreadability in the range of 30.46 ± 0.25 to 36.23 ± 0.61 g cm/s.

The viscosity and consistency are both correlative properties ofgel formulation. Consistency is inversely proportional to the rate of shear. Non-Newtonian flow (shear thinning) is generally preferred, when applied under high shear conditions, as low resistance was observed. Further, the pseudoplastic nature also reduces viscosity decreases, as observed in the formulation. This characteristic property, in turn, defines high spreadability due to the decrease in viscosity and resistance to flow.

### 3.5. In Vitro Release Study

**Figure 6:**
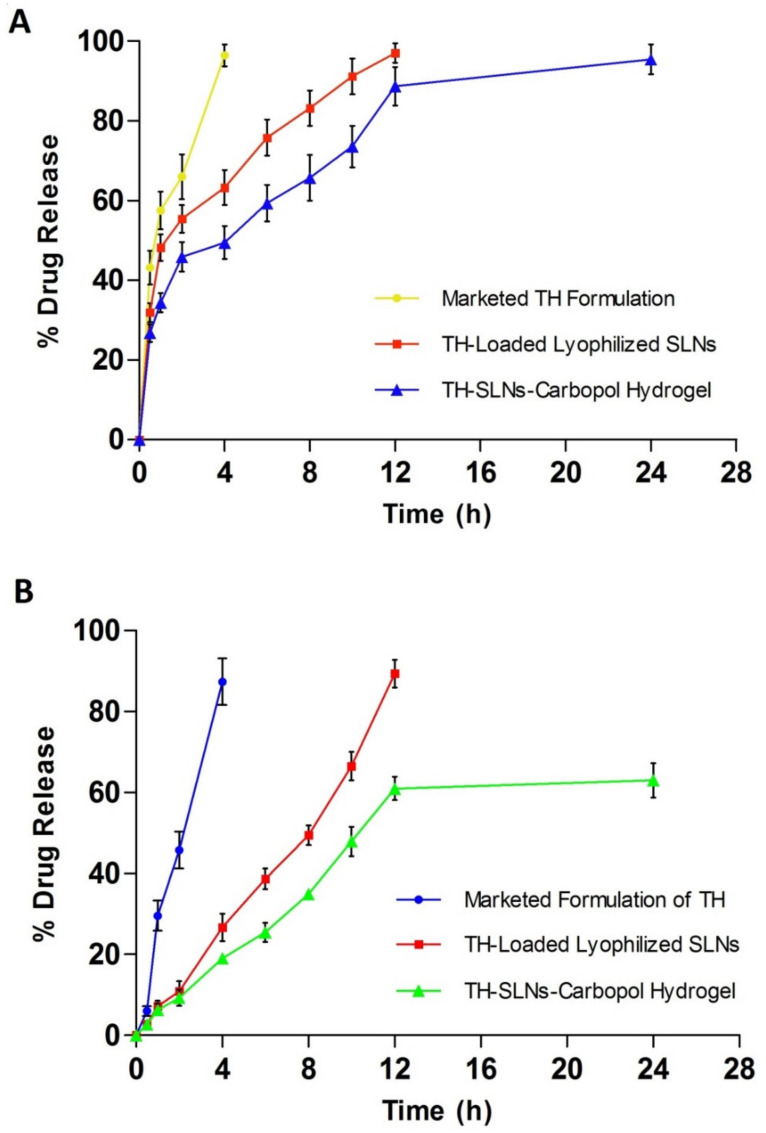
Sustained release of the drug from lyophilized SLNs (**A**) and the TH-loaded carbopol hydrogel over a period of 24 h (**B**).

The in vitro release pattern was determined using a Franz diffusion cell with a dialysis membrane with diffusion media, a phosphate buffer (pH 7.4). It is observed that 50% of the drug was found to release within the initial first 2 h, while, approximately, about 80% of the drug from the TH-loaded SLN-based gel was released upto 12 h (Figure 6A). The SLN-loaded gel showed a 95.47 ± 1.45% cumulative drug release over the period of 24 h (*n* = 6). When compared to conventional marketed TH cream, the SLN-based gel exhibited greater antifungal activity, even at a lower concentration. A Higuchi model of the drug release patternshowed a sustained released pattern of the drug found in the SLN-based gel, over a prolonged period. We observed that approximately 60% of the drug was released over the period of 12 h. The rest of the drug remained in the cutaneous layer of the skin. These studies revealed that SLNs as drug carriers were found to deposit in the corneocytes and thus prolongedthe release of the drug [38].

### 3.6. ExVivo Skin Permeation Studied

We measured the cumulative drug release using rat skin (Figure 6B). We discovered that approximately 60% of the drug was released over a 12-h period. The rest of the drug remained in the cutaneous layer of the skin. The ex vivo drug release data conclude that the SLN-based gel might target the drug to the skin, thus reducing the systemic access of the drug. Ultimately, the systemic side effects can be reduced.

### 3.7. In Vitro Antifungal Activity

**Figure 7:**
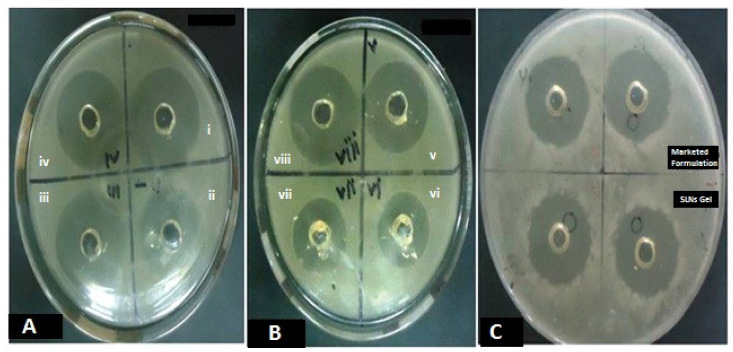
The in vitro antifungal activity of the TH-loaded SLNs carbopol hydrogel, showing significant effect on zone of inhibition compared to the marketed formulation in *Candida albicans* at different time intervals of 24 h on consecutive days for 3 days (**A**–**C**).

In the *Candida albicans* culture, the TH-loaded SLNs gel (0.6%) showed a significant effect on the zone of inhibition when compared with the marketed formulation (Figure 7). The zones of inhibition for the test formulation and conventional marketed formulation were measured to be 34.5 ± 0.69 mm and 27.9 ± 0.49 mm, respectively (*n* = 6). It proves the fungicidal activity of the TH-loaded SLNs gel is comparable to the marketed formulation. The TH shows significant antifungal activity against a broad range of fungi, as stated in previous literature [45]. Whereas when we incorporated TH in the SLN-loaded hydrogel, thein vitroantifungal activity of TH was improved due to the sustained release of TH. The alteration in the release of TH after loading into SLNs leads to its enhanced activity [15].

### 3.8. In Vivo Efficacy of the SLN Hydrogel against C. albicans-Induced Dermal Mycosis in Rats

In vivoefficacy of the test formulation was assessed by the *C. albicans*-induced rat mycosis model. Table 3 showed the number of animals with a positive culture out of seven animals included per group and the log CFU per infected site on rat skin. Isolates of *Candida* were scrapped from the skin and a viability test was carried out. In the TH-loaded SLN hydrogel-treated group, only 2 out of 7 animals exhibited a positive culture test. While 7 out of 7 and 6 out of 7 were found in the control (base formulation) and TH in the ethanol solution group, respectively. In the marketed conventional terbinafine formulation-treated group, only 1 animal showed a positive culture test out of 7. Rapid recovery of infection was observed with marketed conventional terbinafin and TH-loaded SLNs hydrogel formulation-treated groups. The marketed conventional formulation was applied twice a day on the infected site. This topical application of the commercial gel leads to the fast recovery of the infected animals, which may be due to the higher dosing of TH. However, the drawback of the marketed conventional formulations is that they should be applied from time to time; hence, the minimum effective concentration needs to be maintained during the whole therapy. However, the SLN-loaded hydrogel acts as a sustained release system and was successfully used to avoid multiple dosing but shows slower recovery than the marketed conventional topical formulation. Statistical significance of the latter was checked by one-way ANOVA and Dunnett’s multiple comparison test. Significant efficacy of the TH-loaded SLNs hydrogel formulation in treating *Candida* infections when compared to the base formulation (control *p* < 0.05) or TH solution prepared in ethanol (*p* < 0.05) was noted. This result is comparable with that of the marketed conventional terbinafine formulation-treated group, which also showed a significant reduction in log CFU per infected site on rat skin (*p* < 0.01).

### 3.9. Stability Study

The stability study of the optimized lyophilized SLNs showed a slight change in the drug content from 99.7± 0.6 to 98.0 ± 0.4 at room temperature, whereas from 99.8 ± 0.2 to 99.0 ± 0.2 at freezing temperature, as shown in Table 4. Drug leaching from the solid lipid nanoparticles may be the possible reason [46]. This leaching was lower at the freezing temperature than at the room temperature. Particle size analysis also indicated an increase in the size of the nanoparticles from 241 nm to 269 nm over the period of 3 months during the stability study.

The prepared TH-loaded SLNs hydrogel shows a decrease in the drug content after an observation period of 3 months during the stability study. The drug content was found to be 97.1 ± 0.3 and 98.6 ± 0.4 at room and freezing temperatures, respectively, after 3 months, as shown in Table 5. This indicates the preparation being more stable at a cooling temperature than at room temperature. Again, the pH of the TH-loaded SLNs gel was found to decrease over the period from 6.5 to 6.4 ± 0.1.

## 4. Conclusions

The aim of the study was to formulate an SLN-based gel for topical delivery of TH to decrease the dose, dosing regimen, and side effects associated with oral drug delivery. A high-pressure homogenization technique was employed to prepare the TH-loaded SLNs, prepared with the help of glyceryl monostearate as a lipid matrix, Pluronic^®^ F68, a surfactant, and distilled water as a dispersion medium. The drug-to-lipid ratio was optimized to obtain the desired particle size with the highest percent encapsulation efficiency. Desirable results were obtained while preparing the systemic formulation, such as time saving and a reduction in the number of experiments. Cost effectiveness is also important. In this work, an optimized SLN formulation is offered as an alternative to standard formulations for topical treatment of fungal infections, with an improved permeability and reduced dosing regimen and side effects. In comparison to the commercial formulation, the SLN-based gel showed better skin deposition and in vitro antifungal efficacy. This improved TH–SLN-based gel will improve antifungal therapy’s safety, affordability, and tolerance. As a result, developing a topical TH–SLN-based gel could be a unique, industrially scalable, and successful alternative to currently available conventional dosage forms.

## Figures and Tables

**Table 1:** Characterization of the formulated SLNs.

Formulation Code	Particle Size (nm)	Zeta Potential (mV)	Polydispersity Index	% Drug Loading	% Encapsulation Efficiency
P F 1	241.3	−15.2	0.415	6.3602	98.36
P F 2	248.7	−18.1	0.47	4.2192	97.84
P F 3	274.7	−19.4	0.542	3.1652	97.49
P F 4	302.4	−20.2	0.577	2.5322	96.89
P F 5	321.8	−24.8	0.543	2.12	95.39

**Table 2:** Rheological behavior of the TH-loaded SLNs hydrogel.

Viscosity(Cp)	RPM	Viscosity(Cp)	RPM
52,000	10	13,570	100
40,460	20	18,500	60
32,800	30	21,000	50
23,890	50	29,800	30
20,320	60	36,400	20
13,570	100	48,500	10

**Table 3:** Colony-forming unit of *Candida albicans* on skin (dermal mycosis) of rats after treatment with the TH-loaded SLN-based hydrogel.

Treatment	No. of Animals with Positive Culture/Total No. of Animals	Log CFU/Infected Sites
Control (Base formulation)	7/7	5.69 ± 0.45
TH solution in ethanol	6/7	4.01 ± 0.33
TH-loaded SLN-based hydrogel	2/7	2.23 ± 0.19 *^,#^
Conventional marketed formulation of TH	1/7	1.46 ± 0.15 **

* *p* < 0.05, ** *p* < 0.01 vs. control group; ^#^ *p* < 0.05 vs. group received TH solution in ethanol.

**Table 4:** Stability of the terbinafine hydrochloride-loaded SLNs.

	Drug Content		Particle Size
	Room Temperature	Refrigerator Temperature	Room Temperature
Initial	100.0	100.0	241 nm
After 1 month	99.7 ± 0.6	99.8 ± 0.2	248 nm
After 2 months	99.4 ± 0.4	99.7 ± 0.5	250 nm
After 3 months	99.1 ± 0.2	99.5 ± 0.6	269 nm

**Table 5:** Stability of the gel containing terbinafine hydrochloride-loaded SLNs.

	Drug content		pH
	Room Temperature	Refrigerator Temperature	Room Temperature
Initial	100.0	100.0	6.5
After 1 month	99.7 ± 0.5	99.8 ± 0.1	6.6 ± 0.1
After 2 months	98.6 ± 0.7	98.9 ± 0.8	6.7 ± 0.0
After 3 months	97.1 ± 0.3	98.6 ± 0.4	6.4 ± 0.1

## Data Availability

Data is contained within the article.

## References

[1] Trombino S., Mellace S., Cassano R. (2016). Solid lipid nanoparticles for antifungal drugs delivery for topical applica-tions. Ther. Deliv..

[2] Leppert W., Malec-Milewska M., Zajaczkowska R., Wordliczek J. (2018). Transdermal and Topical Drug Administration in the Treatment of Pain. Molecules.

[3] Scorzoni L., de Paula E.S.A.C., Marcos C.M., Assato P.A., de Melo W.C., de Oliveira H.C., Costa-Orlandi C.B., Mendes-Giannini M.J., Fusco-Almeida A.M. (2017). Antifungal Therapy: New Advances in the Understanding and Treatment of Mycosis. Front. Microbiol..

[4] Ud Din F., Aman W., Ullah I., Qureshi O.S., Mustapha O., Shafique S., Zeb A. (2017). Effective use of nanocarriers as drug delivery systems for the treatment of selected tumors. Int. J. Nanomed..

[5] Patra J.K., Das G., Fraceto L.F., Campos E.V.R., del Pilar Rodriguez-Torres M., Acosta-Torres L.S., Diaz-Torres L.A., Grillo R., Swamy M.K., Sharma S. (2018). Nano based drug delivery systems: Recent developments and future prospects. J. Nanobiotechnol..

[6] Jain S.K., Chourasia M.K., Masuriha R., Soni V., Jain A., Jain N.K., Gupta Y. (2005). Solid Lipid Nanoparticles Bearing Flurbiprofen for Transdermal Delivery. Drug Deliv..

[7] Maia C., Mehnert W., Schäfer-Korting M. (2000). Solid lipid nanoparticles as drug carriers for topical glucocorticoids. Int. J. Pharm..

[8] Liu J., Hu W., Chen H., Ni Q., Xu H., Yang X. (2007). Isotretinoin-loaded solid lipid nanoparticles with skin targeting for topical delivery. Int. J. Pharm..

[9] Mei Z., Wu Q., Hu S., Lib X., Yang X. (2005). Triptolide Loaded Solid Lipid Nanoparticle Hydrogel for Topical Application. Drug Dev. Ind. Pharm..

[10] Pople P.V., Singh K.K. (2006). Development and evaluation of topical formulation containing solid lipid nanoparticles of vitamin A. AAPS PharmSciTech.

[11] Souto E., Anselmi C., Centini M., Müller R. (2005). Preparation and characterization of n-dodecyl-ferulate-loaded solid lipid nanoparticles (SLN^®^). Int. J. Pharm..

[12] Souto E.B., Müller R.H. (2006). The use of SLN and NLC as topical particulate carriers for imidazole antifungal agents. Pharm.-Int. J. Pharm. Sci..

[13] Chen H., Chang X., Du D., Liu W., Liu J., Weng T., Yang Y., Xu H., Yang X. (2006). Podophyllotoxin-loaded solid lipid nanoparticles for epidermal targeting. J. Control. Release.

[14] Liu B., Han L., Liu J., Han S., Chen Z., Jiang L. (2017). Co-delivery of paclitaxel and TOS-cisplatin via TAT-targeted solid lipid nanoparticles with synergistic antitumor activity against cervical cancer. Int. J. Nanomed..

[15] Vaghasiya H., Kumar A., Sawant K. (2013). Development of solid lipid nanoparticles based controlled release system for topical de-livery of terbinafine hydrochloride. Eur. J. Pharm. Sci..

[16] Sheu M.-T., Chen Y.-C., Liu D.-Z., Chang T.-W., Ho H.-O. (2012). Development of terbinafine solid lipid nanoparticles as a topical delivery system. Int. J. Nanomed..

[17] Khanna D., Bharti S. (2014). Luliconazole for the treatment of fungal infections: An evidence-based review. Core Évid..

[18] Sudaxshina M. (2004). Drug delivery to the nail following topical application. Int. J. Pharm..

[19] Rarokar N.R., Khedekar P.B., Bharne A.P., Umekar M.J. (2019). Development of self-assembled nanocarriers to enhance antitumor efficacy of docetaxel trihydrate in MDA-MB-231 cell line. Int. J. Biol. Macromol..

[20] Silva A., Gonzalez E., García M.L., Egea M., Fonseca J., Silva R., Santos D., Souto E., Ferreira D. (2011). Preparation, characterization and biocompatibility studies on risperidone-loaded solid lipid nanoparticles (SLN): High pressure homogenization versus ultrasound. Colloids Surf. B Biointerfaces.

[21] Sze A., Erickson D., Ren L., Li D. (2003). Zeta-potential measurement using the Smoluchowski equation and the slope of the cur-rent-time relationship in electroosmotic flow. J. Colloid Interface Sci..

[22] Rarokar N.R., Saoji S.D., Raut N.A., Taksande J.B., Khedekar P.B., Dave V.S. (2015). Nanostructured Cubosomes in a Thermoresponsive Depot System: An Alternative Approach for the Controlled Delivery of Docetaxel. AAPS PharmSciTech.

[23] Novelli F., De Santis S., Diociaiuti M., Giordano C., Morosetti S., Punzi P., Sciubba F., Viali V., Masci G., Scipioni A. (2018). Curcumin loaded nanocarriers obtained by self-assembly of a linear d,loctapeptide-poly(ethylene glycol) conjugate. Eur. Polym. J..

[24] Saoji S.D., Raut N.A., Dhore P.W., Borkar C.D., Popielarczyk M., Dave V.S. (2016). Preparation and Evaluation of Phospholipid-Based Complex of Standardized Centella Extract (SCE) for the Enhanced Deliv-ery of Phytoconstituents. AAPS J..

[25] Silva A.C., Amaral M.H., González-Mira E., Santos D., Ferreira D. (2012). Solid lipid nanoparticles (SLN)-based hydrogels as potential carriers for oral transmucosal delivery of risperidone: Preparation and characterization studies. Colloids Surf. B Biointerfaces.

[26] Benoit S., Afizah M.N., Ruttarattanamongkol K., Rizvi S. (2013). Effect of pH and Temperature on the Viscosity of Texturized and Commercial Whey Protein Dispersions. Int. J. Food Prop..

[27] Harish N.M., Prabhu P., Charyulu R.N., Gulzar M.A., Subrahmanyam E.V. (2009). Formulation and Evaluation of in situ Gels Containing Clotrimazole for Oral Candidiasis. Indian J. Pharm. Sci..

[28] Yong C.S., Choi J.S., Quan Q.-Z., Rhee J.-D., Kim C.-K., Lim S.-J., Kim K.-M., Oh P.-S., Choi H.-G. (2001). Effect of sodium chloride on the gelation temperature, gel strength and bioadhesive force of poloxamer gels containing diclofenac sodium. Int. J. Pharm..

[29] Jenning V., Schäfer-Korting M., Gohla S. (2000). Vitamin A-loaded solid lipid nanoparticles for topical use: Drug release properties. J. Control. Release.

[30] Ankola D.D., Durbin E.W., Buxton G.A., Schäfer J., Bakowsky U., Kumar M.R. (2010). Preparation, characterization and in silico mod-eling of biodegradable nanoparticles containing cyclosporine A and coenzyme Q10. Nanotechnology.

[31] Sanna V., Gavini E., Cossu M., Rassu G., Giunchedi P. (2007). Solid lipid nanoparticles (SLN) as carriers for the topical de-livery of econazole nitrate: In-vitro characterization, ex-vivo and in-vivo studies. J. Pharm. Pharmacol..

[32] Ganeshpurkar A., Vaishya P., Jain S., Pandey V., Bansal D., Dubey N. (2014). Delivery of amphotericin B for effective treatment of Candida albicans induced dermal mycosis in rats via emulgel system: Formulation and evaluation. Indian J. Dermatol..

[33] Deshkar S.S., Bhalerao S.G., Jadhav M.S., Shirolkar S.V. (2018). Formulation and Optimization of Topical Solid Lipid Nanoparticles based Gel of Dapsone Using Design of Experiment. Pharm. Nanotechnol..

[34] Madan J., Dua K., Khude P. (2014). Development and evaluation of solid lipid nanoparticles of mometasone furoate for topical delivery. Int. J. Pharm. Investig..

[35] Freitas C., Muller R.H. (1998). Effect of light and temperature on zeta potential and physical stability in solid lipid nanoparticle (SLN™) dispersions. Int. J. Pharm..

[36] Oehlke K., Behsnilian D., Mayer-Miebach E., Weidler P.G., Greiner R. (2017). Edible solid lipid nanoparticles (SLN) as carrier system for antioxidants of different lipophilicity. PLoS ONE.

[37] Guo D., Dou D., Li X., Zhang Q., Bhutto Z.A., Wang L. (2018). Ivermection-loaded solid lipid nanoparticles: Preparation, characterisation, stability and transdermal behaviour. Artif. Cells Nanomed. Biotechnol..

[38] Padhye S.G., Nagarsenker M.S. (2013). Simvastatin Solid Lipid Nanoparticles for Oral Delivery: Formulation Development and In vivo Evaluation. Indian J. Pharm. Sci..

[39] Andreozzi E., Seo J.W., Ferrara K., Louie A. (2011). Novel Method to Label Solid Lipid Nanoparticles with ^64^Cu for Positron Emission Tomography Imaging. Bioconjugate Chem..

[40] Danaei M., Dehghankhold M., Ataei S., Hasanzadeh Davarani F., Javanmard R., Dokhani A., Khorasani S., Mozafari M.R. (2018). Im-pact of particle size and polydispersity index on the clinical applications of lipidic nanocarrier systems. Pharmaceutics.

[41] Gupta B., Poudel B.K., Pathak S., Tak J.W., Lee H.H., Jeong J.-H., Choi H.-G., Yong C.S., Kim J.O. (2016). Effects of Formulation Variables on the Particle Size and Drug Encapsulation of Imatinib-Loaded Solid Lipid Nanoparticles. AAPS PharmSciTech.

[42] El-Housiny S., Shams Eldeen M.A., El-Attar Y.A., Salem H.A., Attia D., Bendas E.R., El-Nabarawi M.A. (2018). Bendas and Mohamed A. El-Nabarawi. Fluconazole-loaded solid lipid nanoparticles topical gel for treatment of pityriasis versicolor: For-mulation and clinical study. Drug Deliv..

[43] Molan P. (1999). The role of honey in the management of wounds. J. Wound Care.

[44] Janga K.Y., Tatke A., Balguri S.P., Lamichanne S.P., Ibrahim M.M., Maria D.N., Jablonski M.M., Majumdar S. (2018). Ion-sensitive in situ hydrogels of natamycin bilosomes for enhanced and prolonged ocular pharmacotherapy: In vitro permeability, cytotoxicity and in vivo evaluation. Artif. Cells NanomedBiotechnol..

[45] Jessup C.J., Ghannoum M.A., Ryder N.S. (2000). An evaluation of the in vitro activity of terbinafine. Med. Mycology..

[46] Rarokar N.R., Saoji S.D., Khedekar P.B. (2018). Investigation of effectiveness of some extensively used polymers on thermoreversible properties of Pluronic^®^ tri-block copolymers. J. Drug Deliv. Sci. Technol..

